# Impact of Location and Insulation Material on Energy Performance of Residential Buildings as per Saudi Building Code (SBC) 601/602 in Saudi Arabia

**DOI:** 10.3390/ma15249079

**Published:** 2022-12-19

**Authors:** Saleh H. Alyami, Ali Alqahtany, Noman Ashraf, Abdelbagi Osman, Naief Ali Aldossary, Ayman Almutlaqa, Faris Al-Maziad, Maher S. Alshammari, Wadee Ahmed Ghanem Al-Gehlani

**Affiliations:** 1 Civil Engineering Department, College of Engineering, Najran University, Najran 55461, Saudi Arabia; 2 Department of Urban and Regional Planning, College of Architecture and Planning, Imam Abdulrahman Bin Faisal University, Dammam 34212, Saudi Arabia; 3 Department of Building Engineering, College of Architecture and Planning, Imam Abdulrahman Bin Faisal University, Dammam 31451, Saudi Arabia; 4 Chemical Engineering Department, College of Engineering, Najran University, Najran 55461, Saudi Arabia; 5 Department of Architecture, Faculty of Engineering, Al-Baha University, Al-Baha P.O. Box 1988, Saudi Arabia; 6 Department of Architecture, College of Architecture and Planning, Imam Abdulrahman Bin Faisal University, Dammam 31441, Saudi Arabia

**Keywords:** building envelope, thermal insulation, IES-VE, energy simulation, residential building

## Abstract

In hot and humid climates, a significant part of the energy is used to cool the building. There are several ways to reduce this air conditioning load, but one standout is through the selection and design of the right building envelope and its components. The thermal characteristics of the building envelope, in particular the thermal resistance of the insulation used, have an impact on the thermal and energy performance of building structures. Thermal conductivity, which indicates the ability of heat to move through a material given a temperature difference, is the primary factor affecting the performance of a thermal insulation material. Both temperature and humidity changes can affect a material’s thermal conductivity value, which can then change. In fact, due to the fluctuating ambient air temperature and solar radiation, thermal insulation in buildings is susceptible to significant and continuous temperature variations. Thermal insulation used in building walls and roofs helps to reduce the energy demand of the building. It improves thermal comfort and, if used correctly, reduces the operational cost of the building. The present study has focused on the effects of location and insulation material on the energy performance of a residential building by considering five climatic locations in the Kingdom of Saudi Arabia (KSA). Five commonly used insulation materials with different thermal characteristics, namely polyurethane board (PU), expanded polystyrene (EPS), glass wool (GW), urea-formaldehyde foam (UFF), and expanded perlite (EP), were analyzed under various climatic zones as per the Saudi Building Code 601/602. The selected cities were categorized based on cooling degree days (CDD) and outdoor dry bulb temperature (DBT) as hot, very hot, and extremely hot climatic zones. Insulation improves thermal comfort and, if used correctly, reduces running costs. Experiments were conducted to determine the thermal conductivity, and the energy simulation was performed by employing IES-VE software for various insulation options. The findings indicate that the location has a significant impact on the energy performance of the insulating materials. The energy saving potential of polyurethane board (PU) insulation is more attractive in cities with higher DBTs and CDDs than in cities with lower DBTs and CDDs. The benefit of installing insulation ranged from a 2 to 14% decrease in energy demand for the climate zones studied. The sensitivity analysis showed that the energy saving potential of the insulation materials is sensitive to the set-point temperature (ST) band.

## 1. Introduction

One of the most significant energy demands is the energy demand of residential buildings, which has economic and environmental impacts. According to statistics from the International Energy Agency (IEA), the residential sector uses 2142 Mtoe of the 9424 Mtoe total global energy demand [1]. The International Energy Outlook [2] has projected an annual energy consumption growth rate of about 1.9% until 2040. The Middle Eastern region accounted for nearly 6.6% of the world’s total residential energy consumption in 2012. Higher demand for electrical appliances, particularly air conditioners, is blamed for this rise in home energy usage. Energy consumption is always responsible for greenhouse gas emissions, besides other environmental pollution [3]. This consumption is expected to increase further due to the enormous population and economic growth. Compared to other sectors, the building sector consumes around 36% of total energy for cooling, heating, and lighting. Therefore, it is essential to have an approach to decreasing energy usage in buildings [3,4]. It was proposed previously to design buildings engaging in energy-conservative design principles, such as active design that incorporates insulation materials [5,6]. Such a kind of design will reduce energy consumption and lead to cost-saving and a greener environment [7]. Insulation materials such as mineral wool were used in buildings in the USA as early as in the 1880s [8]. In Saudi Arabia, the building sector is dependent on fossil fuel materials for construction and operation. Such behavior is highly destructive and has a negative impact on KSA’s social and economic aspects. However, the principles of sustainable development are more likely to overcome the environmental impact. Buildings that preserve natural resources can be named as green, eco, or efficient buildings [9,10].

An enormous amount of work has been carried out to reduce the impact of energy and natural resource consumption by buildings. Consequently, serious steps were taken to consider the design and operation of future building envelopes [11]. Currently, buildings and their related services in KSA are mostly dependent on conventional ways of design and operation. Thoughtful effort is being made to implement the green building code in KSA [12]. According to Sandineni et al., improving the building envelope can play a central role in energy saving and environmental protection [13]. Such a kind of improvement can be achieved by exploring different key factors such as wall type, thermal mass, insulation, glazing, and roof type. These factors can be observed using building simulation tools (BSTs). Some researchers criticized the BST as a tool for design, testing, and analysis of building components [14]. However, it is also argued that BSTs can be used to support the final design stage. A number of comparative studies concluded that the well-known BSTs are functional and, to varying degrees, integrate with other engineering software [15,16]. Korjenic et al. studied natural insulating materials made from hemp, flux, and jute against traditional insulation materials such as wool and wood. They found that the newer natural insulations showed high thermal capacity, low thermal conductivity, and good sound insulation with the drawback of low resistance against fire and humidity [17,18].

With regard to wall insulation, another comparative study of insulation materials in external walls was carried out by Axaopoulos et al. to investigate the difference between expanded polystyrene, extruded polystyrene, and mineral wool of various thicknesses. Mineral wool was found to be effective when compared with polystyrene insulation [19]. Al-Sanea and co-workers numerically determined the ideal insulating thickness (R-values) for building walls considering the climatic conditions of Riyadh, Jeddah, and Abha. It was found that R-values for Riyadh and Jeddah were optimum in the range of 2.00 to 2.90 m^2^K/W, whereas those for Abha were in the range of 1.34 to 1.99 m^2^K/W [20,21,22]. Krarti [23] used insulated sliding panels to create dynamic blinds that can regulate the solar heat gains of apartment dwelling units in different US climates. It was found that the annual savings ranged from 9.6 to 52.6% in HVAC energy. Serrano-Jiménez et al. [24] experimentally showed that providing polyurethane foam insulation in the carpentry profiles of windows enhanced the thermal performance by 25% and decreased the indoor temperature by 4 °C in buildings in Spain. Zhoa et al. proposed four types of thermal bridges based on their constructional and thermal characteristics with internal insulation of a residential building in the hot summer and cold winter zone of China. They employed the linear thermal transmittance technique in COMSOL software to determine the heat loss through thermal bridges while the parametric optimization was performed by Python. It was found that the annual heating and cooling loads were reduced by 1.3% and 5.7%, respectively [25]. Alwetaishi [26] numerically found the parameters that could impact the energy performance of residential buildings are thermal insulation, shading devices, and window-to-wall ratio in hot regions. Building insulation was found to be among the studied parameters. The research also showed that ASHRAE and CIBSE codes were proven to be inaccurate when used in hot climates.

Densley Tingley et al. have examined the emissions caused by various kinds of insulation materials in buildings and found polystyrene to be the most favorable [27]. Dickson et al. employed IES-VE energy simulation software to study the energy consumption of several insulation materials in cavity walls and reported that cellulosic fiber insulation outperformed other insulations [28]. Long et al. have reported that the thermo-physical properties of insulation materials are very important parameters for energy performance in buildings. The authors found that the heat capacity and thermal conductivity of insulation materials have an opposing effect on energy performance in buildings. Further, they also found that insulation materials have shown preferred energy performance when applied to external walls compared to internal walls of the building [29]. In the same line, a study conducted by Rosti et al. [30] stated that using insulation materials in external walls conserves more energy than internal wall insulation. For roof insulation, it has been found that applying thermal insulation to the roof can save between 2 and 14% of energy. Axaopoulos et al. stated in a previous study that the application of double-glazing sandwiches with gas in the gap can reduce energy costs by about 15 to 20%. Attractively, using thermal insulation in the external walls can save 35 to 60% of energy costs. In addition, they concluded that external wall insulation shows the maximum economic advantage compared to other options. In their study, they also considered the effect of wind directions and direct sun-facing walls in summer and winter [18]. The same idea was also approved by Dickson and colleagues [28]. Alghamdi numerically studied the thermal insulation options for non-insulated buildings and found the savings in annual energy costs are around 16% by the inclusion of insulation into the external wall structure [31]. The optimal R-value for the Riyadh region of KSA is in the range of 2 to 2.9 m^2^K/W, as reported by Al-Sanea et al. [20]. Alrashed and Asif analyzed 16 distinct climates by employing a decision-matrix approach to determine the best climatic classification for building energy modeling in KSA and recommended that KSA could be divided into five separate climatic zones, namely Dhahran, Guriat, Riyadh, Jeddah, and Khamis Mushait [32].

A review of previous research and studies has shown that the thermal properties of insulation materials can change significantly as the operating ambient dry bulb temperature and humidity change. In Saudi Arabia, the cooling design dry bulb temperature typically surpasses 40 °C. The ambient dry bulb temperature and relative humidity (RH) are the significant factors affecting the thermal conductivity of insulating materials [33]. Most thermal insulation studies have focused on material types, thickness, and characterization, with little attention given to the impact of climatic conditions on the energy efficiency of insulation. As a result, a more realistic assessment of thermal insulation performance for Arabian climatic conditions is certainly required for a more accurate assessment of thermal performance and better energy-efficient design. Accordingly, the present study focused on the energy saving potential of insulating materials for different climate zones in Saudi Arabia, categorized according to the International Energy Conservation Code (IECC) and the Saudi Building Code. Consequently, a typical residential building was studied with and without thermal insulation to examine the impact of insulating materials and locations on the energy-saving potential under different climate zones of Saudi Arabia.

## 2. Materials and Methods

### 2.1. Thermal Conductivity Test

**Figure 1:**
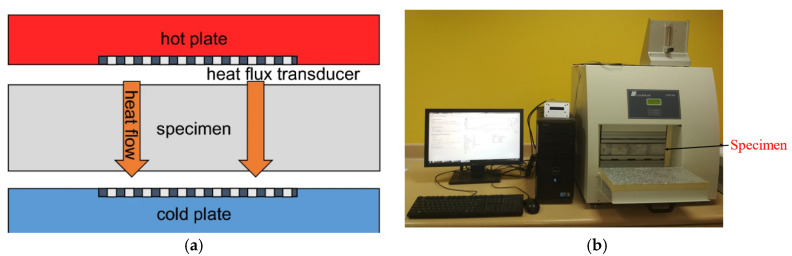
Thermal conductivity experimental setup: (**a**) schematic view; (**b**) pictorial view.

The experimental investigations were conducted to determine the thermal characteristics of the studied insulation materials. The thermal conductivity of the insulation samples were measured under steady-state conditions using the heat flow meter that conforms to ASTM C518, JIS A1412, ISO 8301, and DIN 12667 standards [34,35]. The thermal characteristics of the sample were determined by placing the specimen between the hot and cold plates (temperature gradient) of the heat flow meter. The heat transfer coefficient was calculated from the measured heat flow through the specimen divided by the cross-section area and the applied temperature difference. The heat flow meter provides a rapid and easily determined thermal conductivity value with a high level of accuracy [36]. A schematic diagram and pictorial view of the Linseis Heat flow meter is shown in Figure 1a,b, respectively. The accuracy of the testing machine is about 1.4% of the true value of the thermal conductivity.

### 2.2. Simulation Software and Building Specification

**Figure 2:**
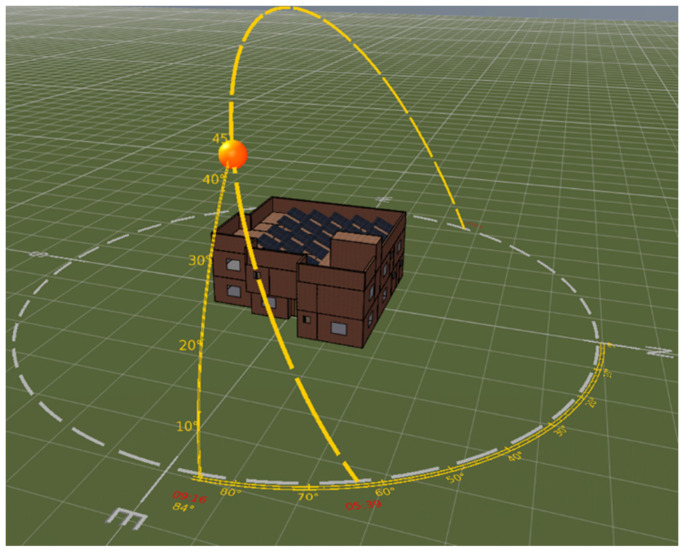
3D model layout of the building in IES-VE.

Building simulation tools have emerged to provide building professionals with a robust way to analyze building energy performance. They still encounter some criticism by many building scientists; these critiques lie in their approximation, flexibility, and integration with other engineering software [16]. The study was conducted on an existing double-storey residential building. The building modeling and simulation were carried out in the IES-VE (Integrated Environmental Solutions—Virtual Environment) energy simulation software. Simulations were performed to evaluate thermal performance of different insulations in five different climate regions of Saudi Arabia. Figure 2 shows the 3D model of the building. IES-VE is a comprehensive building performance analysis software tool used to perform advanced dynamic thermal simulation in time steps. It is designed exclusively for analyzing and investigating the energy performance of a building [37].

**Figure 3:**
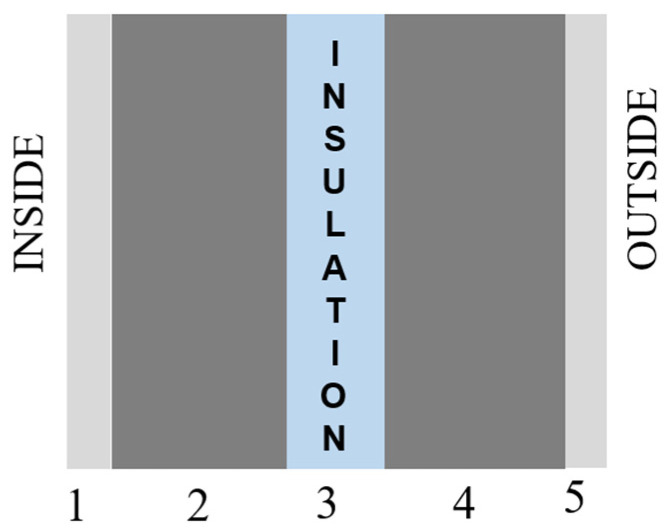
External wall configuration—50 mm insulation (3) sandwiched by 100 mm lightweight concrete block (2 and 4) and 10 mm cement plaster (1 and 5).

The computations are carried out employing a thermal balancing method that accounts for the concurrent interaction of model–building with external environmental factors to be assessed along with numerous loads on an hourly basis. It is simple to use, has a range of modules, and provides a comprehensive analysis of building performance. Several scholars have identified it as one of the most effective simulation software tools and have frequently employed it in their studies and analyses, according to the examined literature. Several researchers have compared the IES-VE simulation results to field observations and validated the software based on the degree of discrepancy in values in the range from 0 to 10% [38,39]. Several academics have therefore demonstrated that IES-VE is an appropriate and reliable simulation tool for research purposes. As a result, IES-VE was used in this study to explore energy performance. Table 1 summarizes the study building’s specifications and physical characteristics. Parameters such as occupancy, equipment power density (EPD), lighting power density (LPD), relative humidity (RH), air speed and infiltration rate, and set-point temperature in compliance with the ASHRAE standard were put into the simulation model. Uninsulated walls, uninsulated roofs, and single-glazed windows were chosen as the basic cases since they are the most common in Saudi Arabia. The exterior wall configuration considered for the study is depicted in Figure 3. Table 2 summarizes the studied insulation options with their abbreviations.

### 2.3. Weather Data and Climatic Classification

The International Weather for Energy Calculations 2.0 (IWEC2), ASHRAE, provided the climate data for the five cities. Climate Consultant software (version 6.0) is a graphic-based computer program developed by the University of California Energy Institute (UCEI), Berkeley, CA, USA. It is used to retrieve the data from the weather file. Table 3 shows the meteorological data for the five cities in Saudi Arabia that were studied along with their elevations [40], cooling degree days (CDD), maximum dry bulb temperature (DBT), etc. The annual CDD was taken from the Saudi Building Code (SBC) 601/602. The values were determined at the base temperature of 10 °C. In terms of cooling degree days, it has been found that all Saudi Arabian cities are cooling-dominated, with the cooling load of buildings outnumbering the heating load [33,34]. According to the Saudi Building Code [35], the Saudi Arabian cities are categorized depending on CDD: extremely hot, very hot, and hot, as summarized in Table 4.

### 2.4. Mathematical Modeling

The first stage in evaluating the building’s energy usage was to identify the various sources, modes, and power-consuming elements. The IES VE simulation takes into consideration the following sources, modes, and power-consuming components:

1. Sensible heat transmission through walls and opaque exterior surfaces (walls, roofs, floors, doors, and ceilings) as well as transparent façade surfaces (glazed windows and skylights). Sensible and latent heat flows from ventilation/infiltration.
2. Heat gains from people, illumination, and machinery that are sensible and latent.
3. Direct power consumption for devices and lighting.

The total energy required is determined as the sum of the cooling load, heating load, lighting load, and device load. The first three items in the list above help reduce the energy requirements of HVAC systems for cooling and heating. In cold-dominated climates, however, the proportion of heating demand to cooling load is relatively small (or negligible). As a consequence, the heating requirement was neglected when assessing the total energy requirement. Furthermore, as it is unaffected by changes in the choice of passive design, the equipment load remained constant throughout the study, while the cooling and lighting loads fluctuated (the lighting load varies when the glazing system changes). The simulation software for the present building used the following set of mathematical equations:

Sensible heat flow by conduction through wall, roof, glass, partitions, ceiling, and floor [41,42]:

SH_cond_ = U × A × CLTD (1)

Sensible heat flow by radiation from external opaque and transparent surfaces:

SH_Radia_ = A × SHGC × SC × CLF (2)

Sensible and latent heat flows by ventilation/infiltration [41,42]:

SH_(vi,s)_ = C_s_ Q∆T (3)

SH_(vi,l)_ = C_l_ Q∆w (4)

SH_(vi,t)_ = C_t_ Q∆h (5)

Air leakage rate Q_i_ = ACH(V/3.6) (6)

Internal sensible and latent heat gains by occupants, lighting, and equipment [41,42]:

H_(ig,s) = 136 + 2.2A_cf + 22N_oc(7)

H_(ig,l) = 20 + 0.22A_cf + 12N_oc(8)

Lighting and equipment load [41,42]:

W_L = LPD × A × t(9)

W_eqp = EPD × A × t(10)

## 3. Results and Discussion

This section summarizes and discusses the main findings of the work. The analysis related to thermal and energy performance of various insulation options was performed by the IES-VS energy simulation software.

### 3.1. Thermal Conductivity

**Figure 4:**
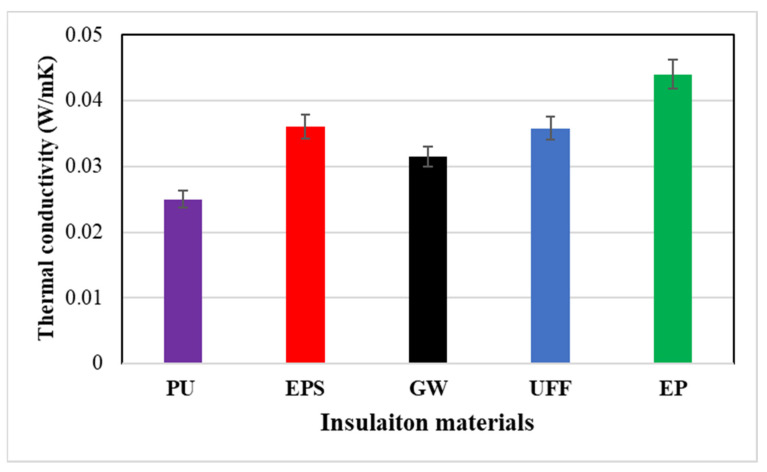
Thermal characteristics of various insulation samples.

The thermal conductivity of the various studied insulation materials was experimentally determined as shown in Figure 4. The observed thermal conductivity values varied between a minimum of 0.025 W/mK for the PU sample and a maximum of 0.044 W/mK for EP insulation. The thermal conductivities of the PU, EPS, GW, UFF, and EP insulations were 0.025 W/mK, 0.036 W/mK, 0.0315 W/mK, 0.0358 W/mK, and 0.044 W/mK, respectively. The determined thermal characteristics of the various insulation options were in line with results reported by other publications in the literature [43,44,45,46].

### 3.2. Effect of Climate and Location on Monthly Energy Consumptions with Various Insulations

**Figure 5:**
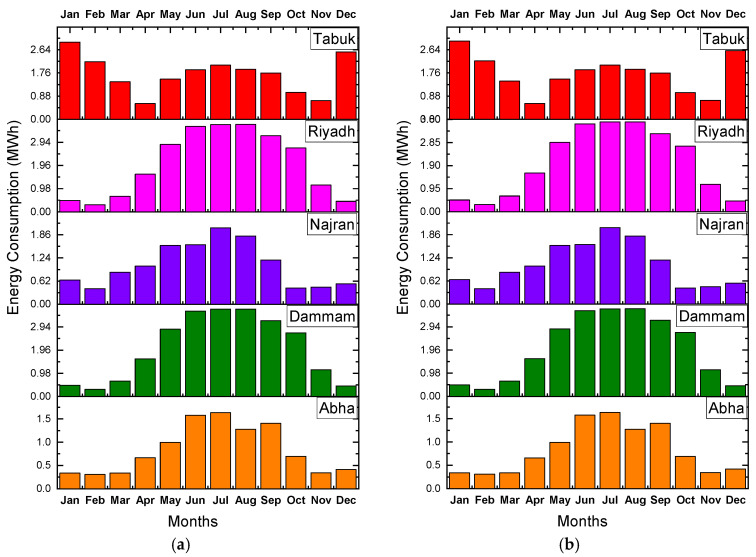

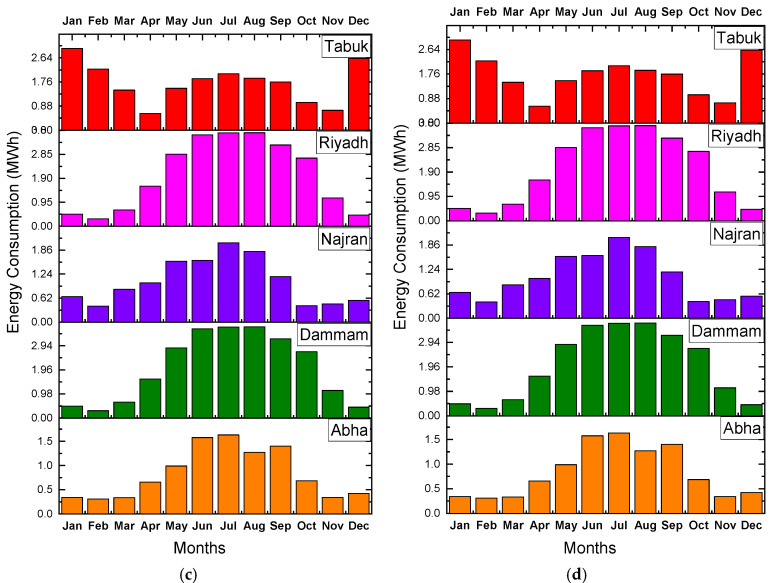
Comparison of monthly energy consumption by various insulation options, for different climates: (**a**) polyurethane board (PU) base case; (**b**) expanded polystyrene (ESP); (**c**) urea-formaldehyde foam (UFF); (**d**) glass wool (GW).

**Figure 6:**
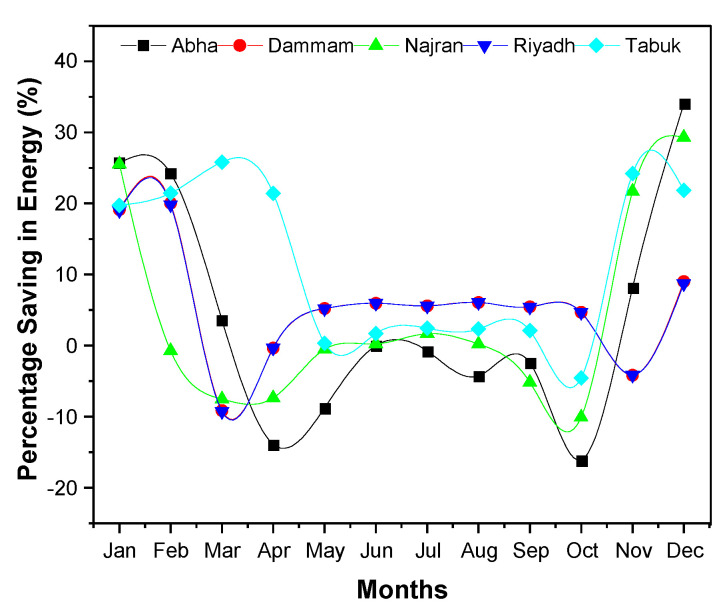
Percentage saving in monthly energy consumption by adding PU insulation for different climates.

Figure 5 compares the monthly energy consumption of the building, considering five different alternatives of building insulation, for the various climatic conditions. The thickness of insulation was in part guided by the data published by Al-Sanea and coworkers, who recommended the optimum thickness of insulation be 50 mm for building walls in KSA [20,22]. The results of the energy consumption of the building, considering five different alternatives of building insulation and climatic condition, are depicted in Figure 5. The addition of insulation to the wall structure reduced the monthly energy demand (Figure 6), as expected, because introduction of insulation reduces the overall heat transfer coefficient (U-value) of the wall, which eventually decreases the heat gain [33,47,48]. The monthly energy requirement almost coincided with the CDD.

**Figure 7:**
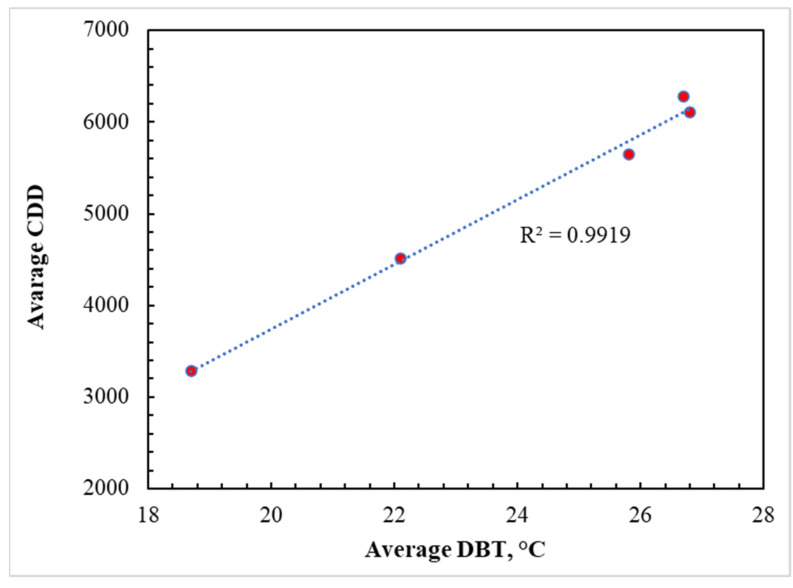
Correlation between DBT and CDD of the studied cities.

The CDD values are direct indicators of the building’s cooling demands, which are around 75 to 80% of the annual energy consumption in KSA. As shown in Figure 7, regression analysis of the correlation among different meteorological data revealed that the DBT and CDD were the best fits to each other, with an R^2^ value of 0.9919. The addition of insulation, irrespective of its type, reduced the energy consumption on a monthly basis as well as the overall annual energy consumption (Figure 6). It is most likely attributable to the remarkably low thermal conductivity (K-value) of insulation, as elaborated in [49,50,51], as a result, the rate of heat transmission through various building components is reduced. However, a close observation of Figure 6 reveals that, for cities in the hot region like Abha, which has the lowest DBT and CDD values, the insulation was not appealing during the warm months as the net fabric heat gain was negative, whereas it was positive for very hot cities (Tabuk) and extremely hot (Dammam and Riyadh) regions. However, when the insulation was added, the heat gain in the hot region (Abha) was still negative, resulting in heat gain [52]. As a result, as shown in Figure 4, the combined impact of internal gain and fabric gain caused excessive heat accumulation by the use of insulation, as evidenced by the increase in energy consumption (negative value for reduction in energy demand). This agrees with the observation of Mujeebu and Ashraf [49], who concluded that, in high-temperature climates, enhanced thermal insulation of the envelope would increase the energy demand in the absence of an alternate strategy to drive the accumulated heat out of the building. For a given building and orientation, the energy performance of both of these options depends on many parameters, such as the climatic location, the share of heating and cooling demands, CDD, the monthly fluctuation of DBT from the indoor set-point temperature, and global horizontal irradiance (GHI). Moreover, the influence of GHI and several other factors such as elevation, latitude, sunshine duration, and humidity on the building’s energy demand was reported by Chen et al. [53]. It can also be observed in Figure 5 that the cities in the extremely hot regions (Dammam and Riyadh) have a higher energy demand during the summer months compared to the winter. This can be explained by the significant increment in heat gain into the building due to the high temperature difference.

### 3.3. Effect of Climate and Location on Annual Energy Consumptions by Insulation Type

**Figure 8:**
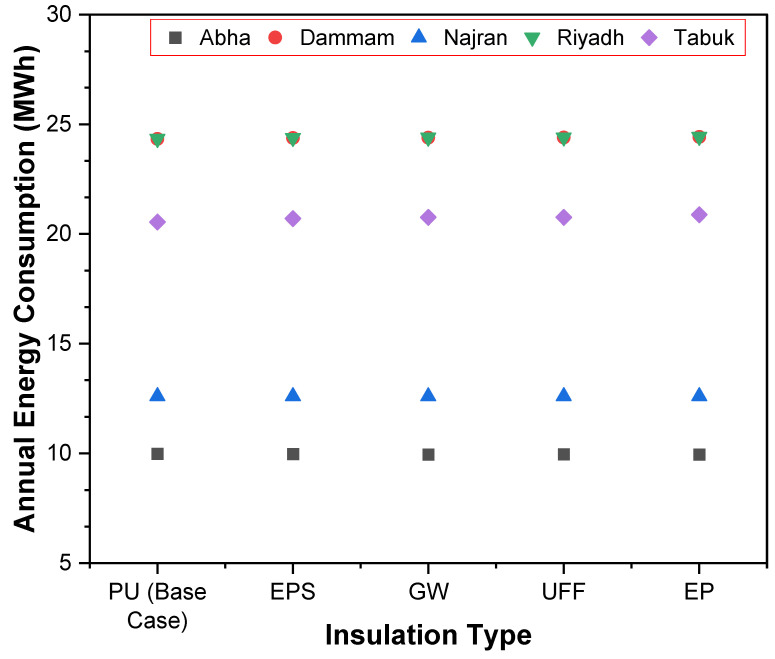
Annual energy demand by insulation type for different climates.

As already described, the respective thermal and energy performances of the five wall structures, consisting of five different types of insulation, were numerically determined. Figure 8 illustrates the annual energy consumption of the building with various types of insulation. The annual energy consumption was observed at 9.9 MWh in Abha, while it was recorded at its highest in Dammam and Riyadh (25.5 MWh). By carefully examining the data, it was found that the annual energy consumption of Dammam and Riyadh follows a similar pattern with various insulation options. This could be explained by the fact that the CDD and DBT for Dammam and Riyadh were almost the same as those of their location, which falls under the extremely hot climatic zone of KSA according to SBC 601 and 602 and International Energy Conservation Code (IECC) [52].

**Figure 9:**
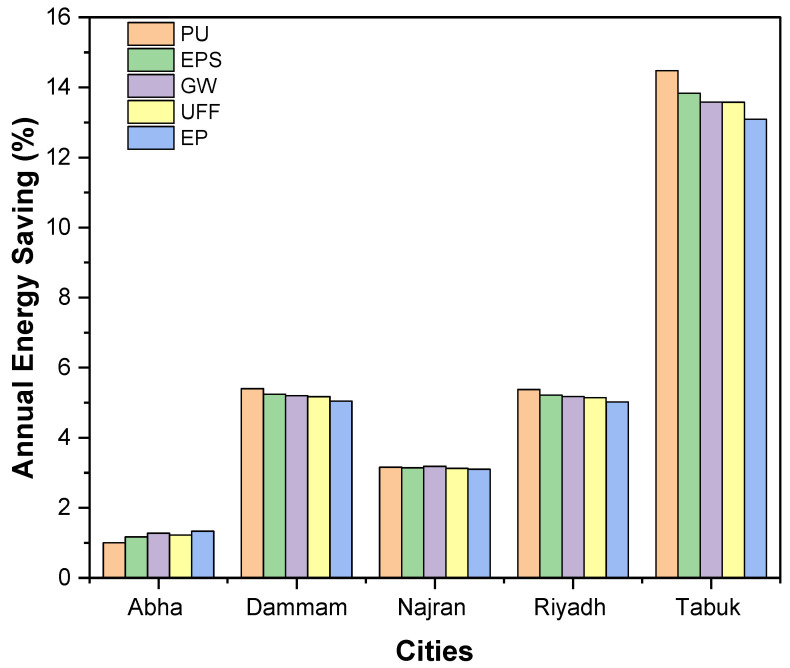
Energy saving potential of various insulation types for different climates.

Figure 9 depicts the percentage savings in the annual energy requirements of the building. The energy saving potential of PU insulation was found to be 5.4% when compared with an uninsulated wall configuration, which was the best among the studied insulation options for the cities that fall under extremely hot regions (Dammam and Riyadh), followed by EPS (5.24%), GW (5.20%), UFF (5.17%), and EP (5.05%). In comparison to the other cities, Tabuk had the maximum insulating effect. The EP insulation slightly outperformed PU insulation for the climatic conditions of Abha (a hot region), even though the thermal conductivity of PU insulation (0.025 W/m°C) was lower than that of EP insulation (0.047 W/m°C). This may be explained by two possible mechanisms: first, in the case of Abha, the difference in indoor and outdoor temperature is minimal, which is significant in the case of cities in extremely hot regions (Dammam and Riyadh). Second, by using superior insulation, the U-value of the wall is drastically reduced, resulting in excess heat accumulation and a higher energy load for the building. This behavior is consistent with the study of Mujeebu and Ashraf, who observed the small reduction in energy demand through the inclusion of superior insulation materials in the building envelope [49].

### 3.4. Indoor Air Temperature

**Figure 10:**
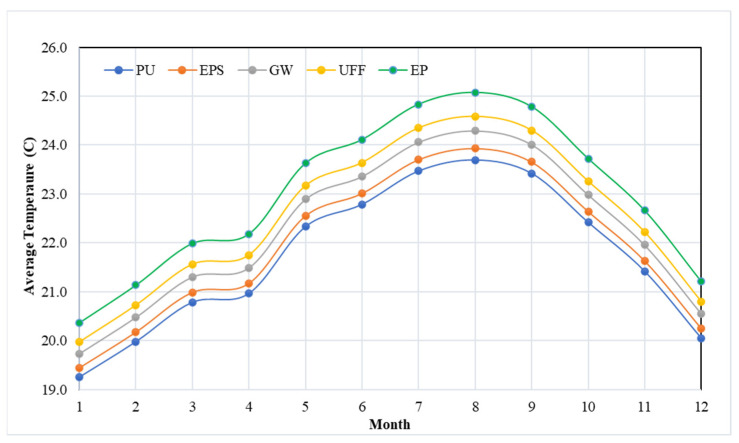
Average monthly indoor temperature variation for various insulation options.

As illustrated in Figure 10, the impact of insulation type on the indoor air temperature of the building structure was assessed for the Dammam climate. The graph clearly demonstrates that August has the highest average indoor temperature, which is ascribed to the high ambient dry bulb temperature. The indoor temperature observed for the wall with UFF insulation was high compared to that of other insulation options, owing to its high K-value (0.358 W/mK). When PU insulation was replaced with EPS, GW, UFF, or EP insulation, the indoor air temperature for the building structure increased by 1.1%, 2.5%, 3.5%, and 4.75%, respectively. This finding may lead to a better understanding of how insulation materials could impact the indoor air temperature, which will eventually affect the thermal comfort of the occupants. Similar findings were also made by Ashraf et al. [38], who demonstrated that selecting a wall with a lower thermal characteristic resulted in a decrease in the indoor temperature of an office building. The results are consistent with the monthly variation in energy demand.

### 3.5. Comparative Analysis between the Insulation Materials

**Figure 11:**
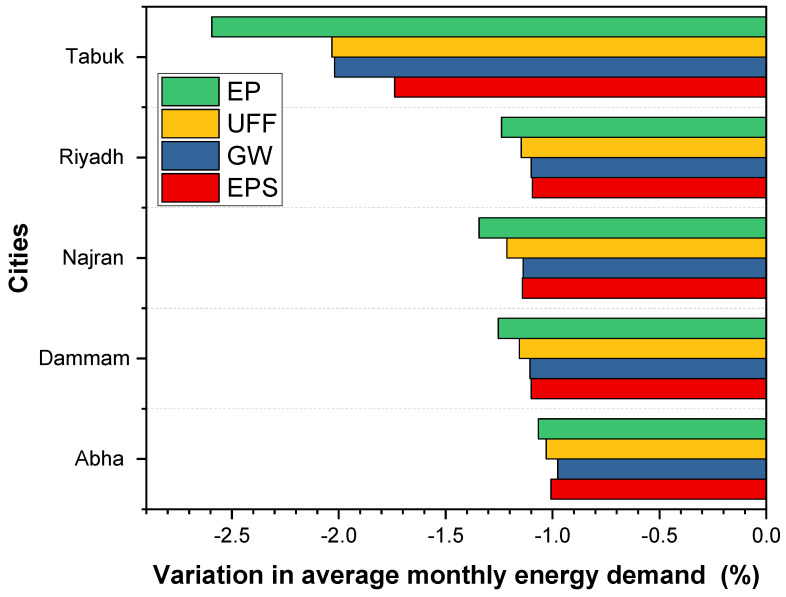
Variation in average monthly energy demand compared to PU base case of the studied cities.

A comparison of the relative variations in energy-saving potential of insulation materials was evaluated. The base case was considered with polyurethane board (PU) insulation. Figure 11 depicts the potential energy savings of various insulation options when compared to the PU (base case). It can be observed that under the extremely hot climatic locations of Dammam and Riyadh, the building energy demand was slightly increased by 1.1%, 1.2%, 1.18%, and 1.3%, respectively, by replacing the PU (base case) insulation with EPS, GW, UFF, and EP, respectively. The optimum insulation was found to be PU (base case) because of its low thermal conductivity (0.025 W/mK), which was the lowest among the studied insulations. In general, replacing PU insulation with other insulation materials increased energy demands as well as total annual energy demand. This can be attributed to the variation in thermal characteristics of the insulation materials [54,55]. A close observation of the comparison reveals that the insulation with a lower K-value is not attractive for Abha. Studies have shown that for moderate climate such as Abha, the internal heat gain is significant, which is due to lighting, equipment, and occupancy, compared to the heat gain via the wall structure. The cumulative effect of internal gain and fabric gain creates excessive heat accumulation by low thermal conductivity insulation materials such as PU, which increases the energy demand of the building [20,49,54,55].

### 3.6. Sensitivity Analysis

The energy saving potential due to the various insulation materials is attributed to the variations in thermal characteristics (as discussed above). There are certain operating conditions that influence the building’s energy demand, such as the set-point temperature (ST) and relative humidity (RH). Accordingly, a sensitivity analysis of these operating parameters was performed for insulation options as well as for the base case. For each option, the annual energy consumption was estimated by varying ST and RH, and the percentage saving with respect to the corresponding baseline energy consumption was obtained in each case for Tabuk region, as summarized in Table 5. It is obvious that while RH had almost negligible impact, ST showed remarkable influence on the energy saving potential of studied insulation options that offer maximum saving in energy for the widest ST range (19–27 °C). A detailed analysis of the crucial influence of ST and its set band on energy consumption has been reported by Mujeebu et al. [49,50,51], who stated that wider ST bands would ensure energy efficient operation of the air-conditioning system. It is worth noting that the ST range set in the present simulation was also 19–27 °C, which is consistent with the mostly accepted temperature range (20–27 °C) for residential buildings according to ASHRAE standards [39]. Thus, it could be deduced that the predicted energy saving potential of insulation materials is independent of RH and is valid for the widely accepted range of ST. A similar pattern of results was observed for other studied regions.

## 4. Conclusions

This paper investigates the effect of location and insulation material type on thermal and energy performance of the buildings in Saudi Arabia. The findings can be summarized as follows:

- The thermal conductivities of the PU, EPS, GW, UFF, and EP insulations were 0.025 W/mK, 0.036 W/mK, 0.0315 W/mK, 0.0358 W/mK, and 0.044 W/mK, respectively.
- The insulated wall, irrespective of the type of insulation, reduced the annual energy demand by around 2 to 14% compared to the uninsulated wall of the building structure. The energy saving potential of the insulation materials significantly differed with the climatic conditions.
- The application of insulation could yield energy saving of 1.2%, 5.2%, 5.15%, 3.16%, and 13.6% in Abha, Dammam, Riyadh, Najran, and Tabuk, respectively.
- A sensitivity study on operational factors including RH and ST was used to validate the estimated energy saving potential and showed that it was relatively independent of RH and valid for the commonly used range of ST.
- The annual average indoor air temperature of the building was found to be 21.7 °C, 21.9 °C, 22.3 °C, 22.5 °C, and 23.2 °C for walls with PU, EPS, GW, UFF, and EP insulation, respectively.
- The results showed that while meeting the code requirement for an energy efficient envelope could result in a significant reduction in cooling energy demand, it actually increases the cooling demand in Abha, which represented hot regions.
- The difference in primary energy was not significant between glass wool (GW) and urea-formaldehyde foam (UFF) insulation.
- The polyurethane board (PU) insulation was found to be the most effective for the cities of extremely hot regions (Dammam and Riyadh), whereas expanded perlite (EP) insulation was efficient in hot climatic zones (Abha).

The outcomes of this research will be useful to engineers, policymakers, and designers, particularly in the application of thermal insulation materials for buildings in different climates of Saudi Arabia. The study could be applied to all locations falling under International Energy Conservation Code (IECC) climate zones 1, 2, 3A, and 3B. Future research could focus on the lifecycle cost (LCC), life cycle assessment (LCA), and indoor thermal comfort of different insulation materials. Different types of buildings, with varied window-to-wall ratios, heating/tropical environments, and other geographical locations are also subjects for additional research.

## Figures and Tables

**Table 1:** Physical and functional properties of the building.

Type of building	Residential building
No. of floors	2 floors
Orientation	North–south
External wall	200 mm CMU, outside 10 mm cement plaster, inside 20 mm gypsum plaster (U-value = 2.17 W/m^2^K)
Roof	12 mm cement roof tiles, 4 mm thick bitumen layer, 50 mm expanded polystyrene, 4 mm thick bitumen layer, 300 mm concrete slab, 20 mm inside gypsum plaster (U-value = 0.45 W/m^2^K)
Glazing	Double-glazed clear low-e 6/12/6 mm (U-value = 1.78 W/m^2^K)
Floor height	4.2 m
Longitude	50.17° E
HVAC system	Package air-conditioning system
Set-point temperature	19 to 27 °C
Occupancy	0.02 person/m^2^
Lighting power density	6 W/m^2^
WWR	1:6.5

**Table 2:** Type of thermal insulation with abbreviation.

Sr. No	Insulation Name	Abbreviation
1	Polyurethane board	PU
2	Expanded polystyrene	EPS
3	Glass wool	GW
4	Urea-formaldehyde foam	UFF
5	Expanded perlite	EP

**Table 3:** Climate data of selected cities of Saudi Arabia.

Sr. No.	Cities	Latitude	Longitude	Climate Classification According to SBC 601/602	Zone According to IECC	Elevation	CDD10	DBT (Max) °C
1	Abha	18.23 N	42.65 E	3	3A and 3B	2093	3289	34
2	Dammam	26.45 N	49.82 E	1	1	12	6274	49.2
3	Najran	17.62 N	44.42 E	1	1	1212	5643	42.7
4	Riyadh	24.70 N	46.73 E	1	1	620	6107	47.6
5	Tabuk	28.38 N	36.60 E	2	2	768	4508	44.2

**Table 4:** Climatic classification under Saudi Building Code (SBC) 601/602.

Climate Classification as per SBC 601/602	Zone as per SBC	CDD 10 °C Range	Climatic Zones
Extremely hot	1	Greater than 5000	Riyadh, Dammam, Najran
Very hot	2	From 3500 to 5000	Tabuk
Hot	3	Less than 3500	Abha

**Table 5:** Results of sensitivity analysis.

Operating Parameter	Saving in Annual Energy Consumption (%) Option				
	PU	EPS	GW	UFF	EP
Relative humidity (%)					
35	14.47	13.83	13.58	13.58	13.09
40	14.47	13.83	13.58	13.58	13.09
45	14.47	13.83	13.58	13.58	13.09
**50**	**14.47**	**13.83**	**13.58**	**13.58**	**13.09**
55	14.47	13.83	13.58	13.58	13.09
60	14.47	13.83	13.58	13.58	13.09
Set-point temperature (°C)					
**19–27**	**14.47**	**13.83**	**13.58**	**13.58**	**13.09**
20–24	16.21	15.49	15.21	15.20	14.66
20–25	15.92	15.21	14.94	14.93	14.40
22–23	15.63	14.94	14.67	14.66	14.14
24–24	16.65	15.91	15.62	15.61	15.05

## Data Availability

Not applicable.

## Abbreviation

SH_cond_	Sensible heat flow by conduction (W); sensible heat flow by conduction
SH_Radia_	Sensible heat flow by radiation (W)
SH_(vi,s)_	Sensible ventilation/infiltration load (W);
SH_(vi,l)_	Latent ventilation/infiltration load (W)
SH_(vi,t)_	Total ventilation/infiltration load (W);
U	Overall heat transfer coefficient of the structure considered (W/m^2^K)
A	Surface area (m^2^)
CLTD	Cooling load temperature variation between the building’s interior and exterior (K)
SHGC	Solar heat gain coefficient
SC	Shading coefficient
CLF	Solar cooling load factor
C_s_	Air sensible heat factor
C_l_	Air latent heat factor
C_t_	Air total heat factor
Q	Ventilation/infiltration air flow rate (L/s)
∆T	Temperature difference across process (K)
∆w	Difference in humidity ratio over the process (kgw/kgda)
∆h	Air enthalpy difference over the process (kJ/kg).
ACH	Air exchange rate (changes/h)
V	Volume (m^3^).
H_(ig,s)	Sensible cooling load from internal heat gains (W)
H_(ig,l)	Latent cooling load from internal heat gains (W)
N_oc	Number of inhabitants
H_(ig,s)	Sensible cooling load from internal heat gains (W)
A_cf	Conditioned floor area (m^2^)
N_oc	Number of inhabitants.
W_L	Lighting load
LPD	Light power density (W/m^2^)
W_eqp	Equipment load
EPD	Equipment power density (W/m^2^)
t	Operating hours (h)
A	Building area (m^2^)
